# Small cell bladder cancer: should we consider prophylactic cranial irradiation?

**DOI:** 10.1590/S1677-5538.IBJU.2018.0242

**Published:** 2019-04-01

**Authors:** Tara Nikonow Morgan, Robert M. Turner, Julian Baptiste, Timothy D. Lyon, Jodi K. Maranchie, Ronald L. Hrebinko, Benjamin J. Davies, Jeffrey R. Gingrich, Bruce L. Jacobs

**Affiliations:** 1 Department of Urology, University of Pittsburgh, Pennsylvania, U.S.A;; 2 School of Medicine, University of Pittsburgh, Pennsylvania, U.S.A

**Keywords:** Prophylactic Surgical Procedures, Urinary Bladder Neoplasms, Carcinoma, Small Cell

## Abstract

**Purpose:**

To describe the clinical characteristics, treatment patterns, and outcomes in patients with small cell bladder cancer at our institution, including those who received prophylactic cranial irradiation (PCI) for the prevention of intracranial recurrence.

**Materials and Methods:**

Patients with small cell bladder cancer treated at a single institution between January 1990 and August 2015 were identified and analyzed retrospectively for demographics, tumor stage, treatment, and overall survival.

**Results:**

Of 44 patients diagnosed with small cell bladder cancer, 11 (25%) had metastatic disease at the time of presentation. Treatment included systemic chemotherapy (70%), radical surgery (59%), and local radiation (39%). Six patients (14%) received PCI. Median overall survival was 10 months (IQR 4 – 41). Patients with extensive disease had worse overall survival than those with organ confined disease (8 months vs. 36 months, respectively, p = 0.04). Among those who received PCI, 33% achieved 5 - year survival.

**Conclusion:**

Outcomes for patients with small cell bladder cancer remain poor. Further research is indicated to determine if PCI increases overall survival in small call bladder cancer patients, especially those with extensive disease who respond to chemotherapy.

## INTRODUCTION

Small cell carcinoma of the bladder is a rare, biologically aggressive neuroendocrine cancer that is locally destructive and disseminates early. Due to the high risk of systemic relapse following surgical resection alone, platinum - based chemotherapy is recommended in the neoadjuvant setting followed by consolidation surgery and /or radiation in those who respond (1, 2). Despite this aggressive approach, outcomes remain poor (3).

There has been growing enthusiasm for prophylactic cranial irradiation (PCI) for those patients who demonstrate a response to initial chemotherapy to decrease the risk of intracranial metastases. Although evidence for this approach is limited in small cell bladder cancer, there is strong evidence supporting its use in patients with small cell lung cancer, 40 – 80% of whom develop brain metastases within 2 years of diagnosis (4-6). Use of PCI in limited stage small cell lung cancer has been well established since the early 1990s. A meta-analysis by Auperin et al. showed a 5.4 percent increase in the rate of survival at three years (5). Phase III studies have also demonstrated that PCI improves overall survival from 13% to 27% in those with extensive stage small cell lung cancer at 1 - year (7). As a result, PCI has become a standard of care in some patients with small cell cancer of the lung. However, it is unclear if the same survival benefit of PCI extends to patients with extrapulmonary small cell carcinoma, where the reported incidence of brain metastases is only 5 – 18% (2, 8, 9).

For this reason, we reviewed our institutional experience with small cell carcinoma of the bladder, including a series of patients who received PCI to investigate its use in this population.

## MATERIALS AND METHODS

### Study Population

Using a Boolean search (keyword - based text search using logical operators such as AND, OR, NOT) of surgical pathology reports within the electronic medical record, we identified patients diagnosed with small cell bladder carcinoma at our institution between January 1990 and August 2015. All patients had confirmed histological diagnosis of small cell cancer of the bladder established by a trained genitourinary pathologist. We further identified patients who received PCI. The decision to proceed with PCI was made at the discretion of the patient's treating physician.

### Outcomes

Patient demographics, stage at presentation, treatment, and outcomes, including intracranial relapse rates and overall survival, were evaluated upon retrospective review of the electronic medical record. Overall survival was recorded from the date of histological diagnosis. Dates of death were obtained from the medical record and a search of the public death records.

The incidence of intracranial relapse was defined by development of a cranial metastasis during the study period. Overall survival was stratified by limited and extensive disease, with limited disease defined as organ confined malignancy (stage T2 or less, N0, M0) and extensive disease defined as stage T3 or greater, or N1, or M1 or greater.

### Statistical analysis

Descriptive statistics were performed by calculating frequencies of categorical variables and the median and interquartile range (IQR) for continuous variables. Intracranial relapse was compared between those patients who did and did not receive PCI using a chi - square test. Overall survival was compared between patients with localized and extensive disease using a Mann Whitney U test. Analyses were performed using SPSS software, version 20 (IBM Corp., Armonk, NY). Statistical significance was defined as p < 0.05 using two - tailed tests. The University of Pittsburgh institutional review board approved the study (PRO15090222).

## RESULTS

Characteristics of the study population are summarized in Tables 1 and 2. Of 44 patients included in the study, 73% were male, and the median age at diagnosis was 77 years (IRQ 67 – 80). One third (33%) were smokers at the time of diagnosis. Only 2 patients (5%) had pure small cell carcinoma, whereas the majority (95%) had mixed histology (a predominance of small cell carcinoma mixed with urothelial carcinoma). One in four (25%) presented with metastasis; forty patients (91%) had stage T2 disease or greater at the time of diagnosis.

**Table 1:** Characteristics of study population.

Characteristics		n=44
Age at Diagnosis, years, (IQR)		77 (67-80)
**Gender (%)**		
	Male	32 (73)
	Female	12(27)
**Smoker (%)**		33 (75)
**T Stage (%)**		
	Tis	1 (2)
	T1	3 (7)
	T2	19 (43)
	T3	13 (30)
	T4	8 (18)
**N Stage (%)**		
	N0/NX	35 (80)
	N1	5 (11)
	N2/N3	4 (9)
**Metastases at Diagnosis (%)**		11 (25)
**Radical Surgery (%)**		26 (59)
	Cystectomy (Ileal Conduit)	18 (41)
	Cystectomy (Neobladder)	5 (11)
	Aborted Cystectomy	1 (2)
	Partial Cystectomy	1 (2)
**Local Radiation (%)**		17 (39)
**Prophylactic Cranial Irradiation (%)**		6 (14)
**Systemic Chemotherapy (%)**		31 (70)
	Neoadjuvant	12 (27)
	Adjuvant	16 (36)
	Both	3 (7)
**1-Year Survival (%)**		23 (52)
**5-Year Survival (%)**		7 (16)
**Overall Survival, median, months (IQR)**		10 (IQR 4-41)

**IQR =** interquartile range

**Table 2:** Characteristics of patients treated with prophylactic cranial irradiation.

Characteristic		n=6
Age at Diagnosis, years, (IQR)		77 (67-80)
**Gender (%)**		
	Male	6 (100)
**Smoker (%)**		6 (100)
**T Stage (%)**		
	Tis	1 (17)
	T1	0
	T2	3 (50)
	T3	2 (33)
	T4	0
**N Stage (%)**		
	N0/NX	5 (83)
	N1	1 (17)
	N2/N3	0
**Metastases at Diagnosis (%)**		2 (33)
**Radical Surgery (%)**		2 (33)
	Cystectomy (Ileal Conduit)	1 (17)
	Cystectomy (Neobladder)	1 (17)
**Local Radiation (%)**		4 (67)
**Systemic Chemotherapy (%)**		5 (83)
	Neoadjuvant	2 (33)
	Adjuvant	2 (33)
	Both	1 (17)
**1-Year Survival (%)**		4 (67)
**5-Year Survival (%)**		2 (33)
**Overall Survival, median, months (IQR)**		30 (IQR 6-100)

Overall, there was a 10.2 - month median overall survival (IQR 4 – 41) and a 16% 5 - year survival. The majority received multimodal therapy, as 59% were treated with radical surgery, 39% with local radiation, 70% with systemic chemotherapy, and 14% with PCI. When analyzed separately, patients with extensive disease had an overall survival of 8 months (IQR 3 – 35) versus 36 months (IRQ 8 – 64) in those with limited disease (p = 0.04). In total, 4 (9%) patients developed brain metastases.

Specific details of care and outcomes of patients who received PCI can be found in Table-3. In the subset of patients treated with PCI, 50% had extensive disease at presentation and there was a 33% 5 - year survival and a median overall survival of 30 months (IQR 6 – 100). One of these six patients (17%) experienced intracranial relapse.

**Table 3:** Disease details and outcomes of patients treated with prophylactic cranial irradiation.

Patient	Agee	Metastasis at Diagnosis	TMN Stage	Pre-Treatment MRI or CTH	Surgery	Type	Chemotherapy	Type	Response to Chemotherapy	Local Radiation	Cranial relapse	Extra Cranial Relapse	Local Recurrence	Survival (mths)
1	79	Yes	T2bNxMx	MRI	Yes	TURBT	Yes	Neo	Yes	Yes	No	Yes	Yes	23.8
2	75	No	T3bN1Mx	MRI	Yes	CxIC	Yes	Neo	Yes	No	No	Yes	Yes	6.5
3	72	Yes	T3NxMx	MRI	Yes	TURBT	Yes	Adjuv	Yes	Yes	Yes	Yes	Yes	4.2
4	70	No	TisN0Mx	MRI	Yes	CxNB	Yes	Neo	Yes	No	No	No	No	77.7
5	78	No	T2NxMx	CTH	Yes	TURBT	Yes	Adjuv	Limited	Yes	No	Yes	Yes	36.3
6	49	No	T2NxMx	CTH	Yes	TURBT	Yes	Neo	Yes	Yes	No	No	Yes	166.4

All patients were male and had a smoking history

**Adjuv** = Adjuvant Chemotherapy; **CTH =** Computerize Tomography of the Head; **MRI =** Magnetic Resonance Imaging of the Head; **Neo =** Neoadjuvant Chemotherapy; **CxIC =** Radical Cystectomy with Ileal Conduit Diversion; **CxNB =** Radical Cystectomy with Neobladder Diversion

## DISCUSSION

Small cell carcinomas of the bladder are aggressive malignancies with a high propensity for early metastasis. In our study, 25% had metastasis at the time of diagnosis and > 90% presented with stage T2 or higher, consistent with prior observational studies (3). Accordingly, 70% of patients were treated with local surgery or radiation combined with systemic chemotherapy and 14% of patients were treated with PCI. The median overall survival was 10 months and the 5 - year survival was 16%. Among those who received PCI, 5 - year survival was 33%.

For many patients with small cell lung cancer, PCI is recommended for prevention of intracranial relapse. Several randomized controlled trials show that PCI decreases brain metastases and increases overall survival in patients with both limited - and extensive - stage small cell lung cancer in those who have a good response to initial chemotherapy (5, 7, 10), with an overall increase in survival at 1 - year from 13 to 27% (7). Meta - analyses have shown a significant decrease in the incidence of brain metastases with a hazard ratio (HR) of 0.48 (95% confidence interval [CI] 0.39 – 0.60) (11), and a decrease in overall mortality by 4.4% in patients treated with PCI (4). Of note, there is one contemporary randomized control trial, which questions the utility of PCI in those with extensive disease; in that trial, there was no survival benefit between groups (12).

It remains unclear if the benefits of PCI in small cell lung cancer can be extrapolated to patients with small cell bladder cancer. Few of the published studies discussing PCI in extrapulmonary small cell have included primary bladder tumors, (8, 13-17) the largest documented series citing only 3 patients (2). Most have discouraged the use of PCI in this population as the incidence of brain metastases is thought to be as low as 5 – 18% (2, 8, 9). A pooled - analysis of prior studies (15) concluded that the incidence of brain metastases was 10.5% (95% confidence interval [CI] 7.5% to 14.1%), which is similar to the findings in our series (9%). However, the risk of brain metastases in small cell bladder cancer patients with extensive disease at presentation (bulky tumors, advanced stage (> T3a), or non - cerebral metastasis) increases to 50% (18). Therefore, patients with extensive disease and a good clinical response to chemotherapy may benefit most from PCI. While further study is needed, the Canadian Association of Genitourinary Oncologist guidelines recommend offering PCI in this group, due to the significant negative impact on quality of life and poor survival following development of cranial metastasis (19).

While PCI may improve functioning by preventing or delaying brain metastasis, there are known side effects. Neurocognitive toxicities may preclude its use in patients where the benefit of the treatment is less clear. Acute toxicities are generally mild and include anorexia, constipation, headaches, and leg weakness. A study by Slotman et al. showed hair loss and fatigue were higher in patients who received PCI, while there was no difference in emotional functioning (20). In early treatment techniques, there were significant neurologic sequelae. However, contemporary PCI has fewer long - term effects and the clinical significance of these effects is unclear. Old age (> 60), concurrent administration with chemotherapy, and high radiation doses have all been associated with the neurotoxicity of PCI (21-23). While self - reported decline of cognitive functioning has been reported (24), a number of prospective randomized control trials in the 1990s showed no clinically significant long - term neurocognitive effects in PCI patients (25, 26). Moreover, baseline neurocognitive function may be impaired prior to PCI in small cell cancer patients, further complicating this research (23). Methods to reduce neurotoxicity are underway, including hippocampal avoidance, which has resulted in durable in - brain control and an absence of neurocognitive decline (27, 28).

The findings of this study must be considered in the context of several limitations. Given the rarity of this disease, our series is limited to a small number of patients. However, our experience adds to the current body of literature describing this rare condition and, to our knowledge, includes the largest series of patients treated with PCI. Second, we did not have a method to study cognitive or quality - of - life effects of PCI in our cohort and therefore cannot comment on the tolerability or side effects of this treatment. Third, patients were primarily male and treated at a large academic medical center. Therefore, these results may not be generalizable to other populations. Lastly, not all patients in the study had baseline MRIs. Thus, some patients may have had occult brain metastasis at the time of presentation and may be incorrectly categorized.

Though the benefits of PCI in patients with extrapulmonary small cell carcinoma have been questioned, there may be benefit in select patients, particularly those who present with extensive disease and a high risk of brain metastasis who show an initial response to chemotherapy. Further study is warranted to examine both the benefits and neurocognitive risks of PCI in this population.

## CONCLUSIONS

PCI is now standard of care in some small cell lung cancer patient populations where it has been shown to increase overall survival. We present the largest documented cohort of small cell bladder cancer patients who were treated with PCI. Further research is indicated to determine if PCI may also increase overall survival in small call bladder cancer patients, especially those with extensive disease who have a response to chemotherapy.
